# Improvement of the Surface Layer Properties of 316L Stainless Steel Produced by DMLS Through the Use of a Shot Peening Process

**DOI:** 10.3390/ma19071293

**Published:** 2026-03-24

**Authors:** Kazimiera Dudek, Dominika Grygier, Lidia Gałda

**Affiliations:** 1 Institute of Materials Engineering, Faculty of Exact and Technical Sciences, University of Rzeszow, 35-310 Rzeszow, Poland; kdudek@ur.edu.pl; 2 Department of Vehicle Engineering, Faculty of Mechanical Engineering, Wroclaw University of Science and Technology, 50-370 Wroclaw, Poland; dominika.grygier@pwr.edu.pl; 3 Faculty of Mechanical Engineering and Aeronautics, Rzeszow University of Technology, 35-029 Rzeszow, Poland

**Keywords:** direct metal laser sintering, residual stresses, surface topography, shot peening, anisotropy, 316L stainless steel, additive manufacturing, microstructure

## Abstract

Additive-manufactured (AM) 316L stainless steel, produced via direct metal laser sintering (DMLS) and characterised by a surface topography of high irregularities and tensile residual stresses with specific anisotropy, was subjected to milling and shot peening. The milling process caused a reduction in surface topography parameters, but tensile residual stresses increased significantly. The shot peening process was carried out according to the full factorial design 3^2^ and technological parameters such as a shot diameter in the range of 1-3 mm and an air supply pressure between 0.2 and 0.6 MPa. As a result of the experiments and the analysis, reduced surface topography was achieved, and a favourable residual stress state was formed with compressive stresses. The mechanism of the changes was demonstrated via microstructure observation and statistical models obtained by mathematical analysis.

## 1. Introduction

Direct metal laser sintering is one of the methods of additive manufacturing that enables the production of metallic elements from metal powders. The products are built layer by layer, with the laser beam scanning the surface of the powder layer and sintering the selected regions. Due to the specific method of the desired element’s production, additive manufacturing is a very promising technique that has really slight limitations in the shape and size of fabricated parts. These capabilities enable the producer to form sophisticated and customised parts that improve the performance of the final object for the user. Additive manufacturing techniques have found applications in several industrial sectors such as aerospace, automotive, marine, or biomedical. Different powders of metals are sintered via the application of the DMLS process e.g., alloys of nickel, titanium, or aluminium and also stainless steels. The common stainless steel 316L has found many applications in industry sectors due to its favourable characteristics connected with great corrosion resistance, high strength, and biocompatibility and is also fabricated by additive manufacturing [1]. The parts fabricated with the DMLS technique have quite high density, and the porosity may be controlled with the process parameters. 316L stainless steel can be sintered to obtain high porosity with a large specific surface area. To improve mechanical performance and corrosion resistance, Liu et al. [2] used the nitriding process successfully for porous 316L stainless steel.

Although there are many advantages of the DMLS process, there are also some issues connected with this method [3,4] that need to be solved, or the effects should be minimised. Problems concern the microstructure, mechanical properties, directionality of properties, or repeatability of characteristics. To overcome some defects of the fabricated parts, high-quality metallic powders should be used, and the process parameters should be optimised according to requirements, or new developments should be implemented. Ahmed N. et al. conducted a deep review of the selection of process parameters and the optimisation process according to different criteria and methods for the fabrication 316L stainless steel with the application of laser power bed fusion [5]. The authors described areas of parameter range, problems that may occur, and some solutions and suggested using artificial intelligence and machine learning methods to assist in the optimisation process to accelerate the results analysis. Additive manufacturing enables the production not only of the homogeneous structure, consisting solely of a specific metallic material, but also of the construction of gradient layered structures. According to Yang W. et al. [6], the construction of soft (316L) and hard (WC/316L) layers allows for the generation of higher back stress in the soft layers before the hard layers, leading to the achievement of synergy between tensile strength and elongation. Keller et al. [7] examined hybrid additive manufacturing of 316L stainless steel, where laser wire-directed energy deposition was combined with conventional cold-rolled and annealed 316L steel and deposited on 316L fabricated by laser powder bed fusion. The described results demonstrate that laser wire-directed energy deposition may be effectively combined with laser powder bed fusion. The tensile strength of such hybrid additive manufacturing with laser application was superior to a hybrid consisting of conventional substrate and wire-directed energy deposition. The researchers proposed specific techniques to fulfil the quality standards or to be more precise with the measurements results. As the properties of L-PBF components may differ pointwise, Giovani M. et al. worked out the method of a performance-line instrumented indentation test to assess the mechanical performance with good reliability [8].

It is also expected that after the DMLS process, no more operations are necessary. This expectation is difficult to achieve because during the process, the particles of metallic powder are melted, and then re-solidification occurs when the component becomes the shape that was designed. Everything is associated with high temperature and high temperature change (gradient), so there is a risk that because of the heat transfer mechanism, material shrinkage may not proceed perfectly, causing high residual stresses, pores, or other defects. The important factors that influence the quality of the fabricated parts with the DMLS process are the microstructure and the roughness of the surface. Surface irregularity may be a crucial parameter in the case of required dimensional accuracy and some surface topography functions. Zhang et al. [9] proposed two treatments after 316L fabrication that finally led to improvements in the surface roughness and mechanical characteristics. The first is the deep cryogenic treatment that introduces nanotwins and increases the interplanar spacing; the second is the shot peening process. This procedure allowed for reducing the surface roughness by over 60% and increased the yield strength over twice.

As laser scanning speed significantly influences the 316L stainless steel microstructure and the surface roughness, the manufacturing process run with low laser speed (700 mm/s) allowed for obtaining fine grains that positively affected the tensile strength. With the low speed of the laser, the lowest surface roughness (Ra = 3.31 µm) was also achieved. Due to the collective influence of surface layer parameters, the corrosion and fretting tribocorrosion behaviour was improved, resulting in the lowest friction coefficient and the smallest wear [10]. The influence of surface texture is significant on electrochemical corrosion performance. Bozkurt Y.B. et al. [11] ran some tests with untreated samples and with specific square or circle dimples from 316L and found that the best corrosion resistance occurred for the case with 5% coverage of circle dimples on 316L stainless steel fabricated by selective laser melting. The material losses of this sample with the dimple area coverage were the smallest compared to other textured samples, and the surface damage was similar to that of the untextured one. M. Kořenek et al. [12] investigated the oxidation behaviour of additively manufactured 316L stainless steel after different surface treatments. They found that the rough surface (Sa of 5.33 µm) that was fabricated by selective laser melting and then sandblasted oxidised rapidly. The ground surfaces, which were also subjected to annealing at 1000 °C, were characterised by greater concentration of iron oxides that could lead to deep corrosion. DelRio F.W. et al. [13] carried out deep research on the corrosion behaviour of 316L stainless steel produced by the laser powder bed fusion method. The authors found that the smoothest and densest area with the densest oxide layer was characterised by great resistance to passive film breakdown during the tests. On the other sample with high roughness and with porous parts, a large number of pitting signs were observed.

The built orientation strongly affected the mechanical properties and corrosion resistance of 316L stainless steel manufactured by laser powder bed fusion. According to Jinlong L. et al. [14], samples of LPBF 316L cut perpendicular to the built direction showed lower ultimate tensile strength and yield strength than those cut parallel to the built direction. Samples in the built direction were characterised by better corrosion resistance compared to those in the normal direction that were tested in borate buffer and also sodium chloride solutions. The authors explained this phenomenon by larger grain size and densely packed crystallographic faces in the built direction of 316L stainless steel during laser powder bed fusion. Edge and flat built elements were characterised by higher tensile and yield strengths than those vertically built [15]. The authors also pointed out that the surface roughness of the as-built elements with the L-PBF process acted as the stress concentrator for the fracture initiation, and this factor depended on the orientation. Dixit S. et al. [16] examined the mechanical anisotropy of the additive manufactured 316L stainless steels, taking into account two parameters, for example, scan rotation angle and built orientation. The greatest yield strength, ultimate tensile strength, and ductility were obtained for samples sintered in an orientation along the x-direction and with scan rotation angles between successive layers of 67°. Those samples were characterised by the highest dislocation density, which came from the large residual stresses.

The machining process of materials fabricated by additive manufacturing methods is more challenging than that of conventionally produced metals. The friction coefficient is greater during the machining of laser-melted steel than the standard ones. The machining affected the surface layer of the laser-sintered materials more deeply than the wrought ones, and due to the anisotropic microstructure, the changed surface layer thickness in the laser-sintered materials is usually larger in the built plane [17]. The microstructure and locations of surface features of the selective laser-melted 316L stainless steel significantly influence the surface finish after the electropolishing process. Shen M. et al. [18] studied the mechanism of material removal during electropolishing and found that the roughness of the 316L stainless steel fabricated with additive manufacturing depends on the unsintered powder and sintered area. Shot peening treatment influenced the surface layer parameters, which led to greater wear resistance of additively manufactured 316L stainless steel. 316L stainless steel manufactured by metal injection moulding was subjected to a severe shot peening process, with the application of media in sizes between 300 and 500 µm supplied under a pressure of 0.7 MPa. The time exposure was only 30 s, but it was enough to modify the surface and surface layer. The surface roughness, expressed by the Ra parameter, increased slightly, but the nanocrystalline surface was formed with α’-martensite and mechanical twins. The microhardness of the shot-peened surfaces was greater than that of the as-sintered surfaces, and the wear volume was lower compared to the just-sintered samples at loads of 10 N and 20 N. At a higher load (30 N), the wear of not- and shot-peened AM 316L stainless steel was similar [19]. The detailed nanocrystallisation mechanism of the selective laser-melted 316L stainless steel processed by shot peening was investigated by C. Zhang et al. [20]. The authors used steel shots with a diameter of 0.6 mm and processed the surface at an air supply pressure of 0.4 MPa for a quite long time of 45 min. The deformed surface layer was deep and reached approximately 220 µm. The microstructure of SLMed 316L stainless steel after shot peening was characterized by five dissimilar refinement layers, among others, with an elongated ultrafine grain, short rod-like ultrafine grain, or equiaxed nanograin layer. The authors pointed out that the main mechanism of surface nanocrystallisation was due to two factors occurring: the first was dislocation movement, and the second was twinning segmentation. T. Gundgire et al. [21] applied severe shot peening and reduced the surface roughness of selective laser-melted austenitic stainless steel 316L over twice. Tensile residual stresses were changed into favourable compressive stresses even after one pass of shot peening treatment. Although the plastic deformation observed in the microscopic images reached about 10–15 µm, the mechanical characteristics, such as increased microhardness or compressive stresses, were more than 200 µm in depth. Al-Zuhairi et al. [22] obtained a significant reduction in surface roughness of 316L stainless steel fabricated by powder bed fusion laser beam metal due to the shot peening process. The thickness of the plastically deformed zone observed in optical micrographs was about 15 µm, but the real surface layer changed by shot peening reached approximately 200 µm, which was expressed by an increase in microhardness and compressive residual stresses. The shot peening process influences surface roughness significantly and may have a positive effect on coating adhesion, but in the case of plasma nitriding on 316L stainless steel, Biehler et al. [23] found a detrimental effect on the corrosion resistance. The surfaces of the steel samples were shot peened with quite great intensity with the application of glass shots of diameters in the range of 100–200 µm and an air supply pressure of 3–4 bars. Zhang X. et al. [24] found the optimal parameters for shot peening and TiAlCuN coating and that the cavitation erosion resistance of selective laser-melted 316L stainless steel exhibited the best performance. The surface roughness due to shot peening was reduced by 50% in comparison to as-fabricated 316L steel, and also, the defects were reduced. Surface depressions built on the additive-manufactured samples may limit secondary cavitation and enhance the cavitation erosion resistance, but the size of the depression should be adequate to the load. Summarising the literature review, one may conclude that the additively manufactured metal elements are characterised by almost no limits in the final product shape that may be fabricated very quickly, but still, there are some issues with the microstructure, surface roughness, or residual stresses and features’ anisotropy that may all influence the mechanical properties and the operating characteristics, especially under high loads or in unfavourable environments.

The purpose of this study was to analyse the surface layer characteristics of AM 316L stainless steel after milling and additional shot peening with different parameters. The milling process reduced the surface irregularity after powder laser melting, and shot peening improved the residual stress state but also reversed the surface height that was obtained due to milling.

## 2. Experimental Details

### 2.1. Material and Technology

**Figure 1:**
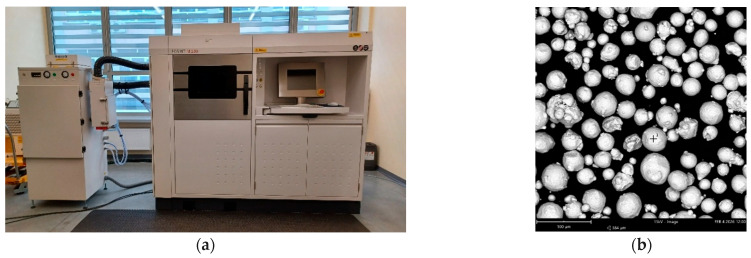
System EOSINT M280 for DMLS (**a**) and SEM image of particles of 316L powder (**b**).

The test samples were built from the metal powders of the stainless steel 316L with the use of the EOSINT M280 system (Figure 1a). 316L stainless steel is characterised by high corrosion resistance and the absence of substances leached in cytotoxic concentrations. The DMLS technique was applied with a laser power of 200 W, laser speed of 1080 mm/s, hatch distance of 0.09 mm, laser spot size of 100 µm, and layer thickness of 20 µm. Argon was used as the protective atmosphere. The maximum particle size of the metal powder was 63 µm (Figure 1b).

**Figure 2:**
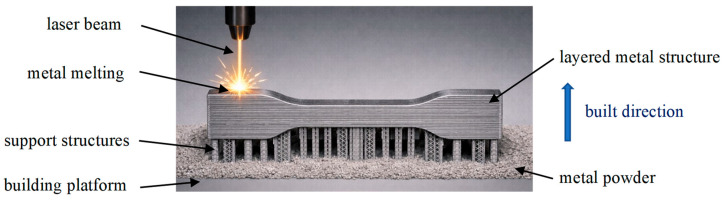
Scheme of the AM sample of 316L stainless steel with the application of the DMLS technique (ChatGPT, GPT-4o).

Components were built layer by layer; once the selected layer was laser scanned, a fresh portion of metal powders was placed, and the sintering process was repeated until the final path was finished. The scheme of the additively manufactured sample is presented in Figure 2 (ChatGPT, GPT-4o was used to generate the scheme).

The external dimensions of the sample were 110 mm × 10 mm × 4 mm. The chemical composition of 316L stainless steel after the DMLS process is presented in Table 1.

**Figure 3:**
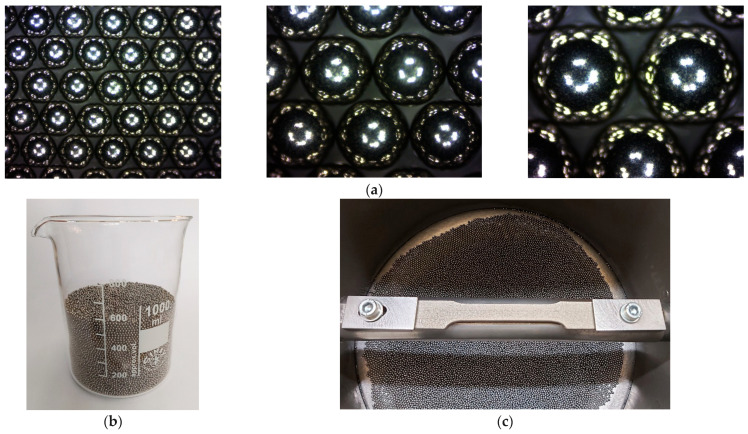
Photos of the shots of d_s_ = 1, 2, and 3 mm (**a**); shot volume of 600 mL for shot peening process (**b**); and the sample mounted in the holder of the stand (**c**).

The front surface of the sample was subjected to the milling process and then to the shot peening process in nine series according to the design of the experiment with two process parameters at three levels each. To examine the potential of the shot peening process after milling, the milling process was realised with the head of multi-plates for stainless steel to introduce an unfavourable stress state. The parameters of the milling process were as follows: rotational speed = 400 rot/min, feed = 0.1 mm/rot, and depth = 0.1 mm. The plan of nine experiments with the shot peening process according to the full factorial design 3^2^ is presented in Table 2. The shot peening process was realised with steel shots of a diameter in range of 1–3 mm that hit the machined surface under the influence of an air supply pressure in the range of 0.2–0.6 MPa. The shots used in the shot peening process and the sample mounted in the stand holder are shown in Figure 3. The distance from the nozzle to the workpiece was constant and equal to 110 mm. The time of the shot peening process was also a constant parameter for each series and amounted to 180 s. The batch of steel shots was the same for each variant, and the volume of the shot batch was 600 mL. The range of technological parameters for shot peening was assumed based on the previous experiments. Also, the processing time as a constant parameter was taken from the authors’ earlier studies, and the criterion in shot peening was to cover the whole processing surface with the machining marks evaluated with the optical microscopy.

### 2.2. Measurement and Analysis Techniques

The microscopic observations were carried out using a 3D digital microscope (Keyence VHX-X1 Series, Mechelen, Belgium) at magnifications ranging from 100× to 2000×. Detailed microstructure examinations were performed using a scanning electron microscope (Phenom XL, Thermo Fisher Scientific Inc., Waltham, Massachusetts, USA) at magnifications from 1000× to 10000×. During the SEM investigations, accelerating voltages of 15 kV and 25 kV were applied. Observations were conducted in material contrast mode using both secondary electron (SE) and backscattered electron (BSE) detectors. The specimens for metallographic analysis were prepared by sectioning material from the central region of the printed samples. The samples were mounted and subsequently subjected to standard mechanical grinding and polishing procedures. The final microstructure revelation was performed by electrolytic etching in oxalic acid. Microscopic observations were conducted on both unetched and etched specimens in transverse and longitudinal sections relative to the built direction. The evaluation of the thickness of the strengthened layer was based on detailed SEM microstructural observations and was further supported by microhardness measurements.

The surface topography was measured with the stylus profilometer 3D i-Series PRO application. The gauge range of the instrument was 2 mm, and the vertical resolution was 2 nm. The measured area was 2 mm × 2 mm. The surface topography was analysed after each technological process, and the surface roughness was assessed for some selected variants. Metrology 4.0 software enabled 3D mapping visualisation, the surface parameters calculation according to the ISO 25178-2:2012, and functional analysis of the surface.

**Figure 4:**
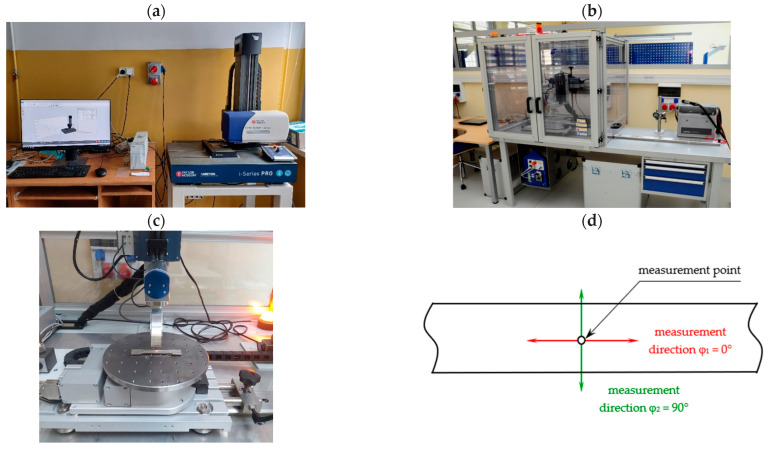
Photos of the 3D stylus profilometer (**a**); X-ray stress analyser (**b**); zoomed-in image of the sample during residual stresses measurement (**c**); and scheme of measurement directions φ_i_ for the example point (**d**).

The residual stresses were measured on the front surface in two orientations: perpendicular to the built direction (φ_1_ = 0—horizontal) and along the built direction (φ_2_ = 90°—vertical) of AM samples. Residual stresses were measured using X-ray stress analyser Xstress 3000 G3 (Stresstech Oy, Vaajakoski, Finland). The measurements of the residual stresses were realised with the *sin^2^ψ* method. The range of the standard symmetrical side inclination psi (ψ) is from −45° to +45°, and the maximum psi (ψ) range is −60° to +60°. During the residual stress measurements, the detectors in the goniometer G3 were at ψ positions: −45°, −37.7°, −30°, −20.7°, 0°, 0°, 20.7°, 30°, 37.7°, and 45°. A collimator that provided a 5 mm spot size was used. The distance between the collimator and the analysed surface was 16.49 mm, and between the detector and the measured surface, it was 75 mm. The exposure time was equal to 15 s, which enabled the collection of data on network constants from the grains with differently arranged diffraction planes. For calculation and visualisation of the results of the residual stresses, XTronic software was used. The residual stress values were measured in all series after DMLS, milling, and shot peening (9 variants). The number of repetitions was equal to three. In Figure 4, the 3D stylus profilometer and the X-ray stress analyser with the scheme of measurement directions φ are presented.

## 3. Results and Discussion

### 3.1. Microstructure Analysis

**Figure 5:**
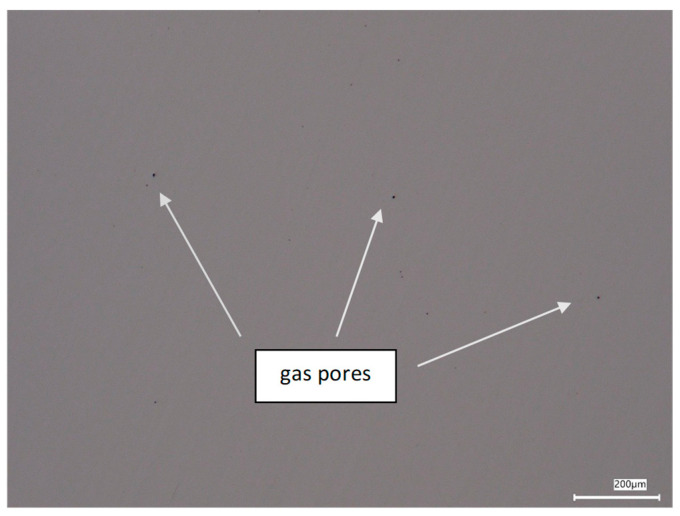
Unetched microstructure of DMLS-processed 316L stainless steel showing small randomly distributed gas pores.

The microstructure of the additively manufactured 316L stainless steel samples was examined using optical microscopy on metallographic cross-sections in the unetched condition. The observations were carried out to evaluate the homogeneity of the material and the level of porosity after the DMLS process. The optical micrographs revealed a relatively dense and homogeneous structure, typical for austenitic stainless steel produced by powder bed fusion. In the unetched state, only a small number of discontinuities were observed (Figure 5). The detected pores were uniformly distributed and exhibited a predominantly spherical morphology.

Quantitative image analysis showed that the total porosity of the material was approximately 0.033% ± 0.015 vol.%, which is consistent with the low porosity levels reported for high-density DMLS 316L components [25]. Such a low defect content confirms the proper selection of processing parameters and the high quality of the fabricated material. On the basis of their regular, near-spherical shape, the observed discontinuities were identified as gas pores. Their formation is directly related to the use of argon as the protective atmosphere during the DMLS process. Because of the very low solubility of argon in both molten and solid stainless steel, the gas present in the melt pool cannot fully escape during the extremely rapid solidification typical of laser powder bed fusion. As a result, gas bubbles become trapped within the solidifying material, forming spherical pores in the microstructure [25,26]. Similar pore formation mechanisms associated with complex laser–powder interactions and rapid cooling have been widely reported for SLM-processed 316L stainless steel [27].

**Figure 6:**
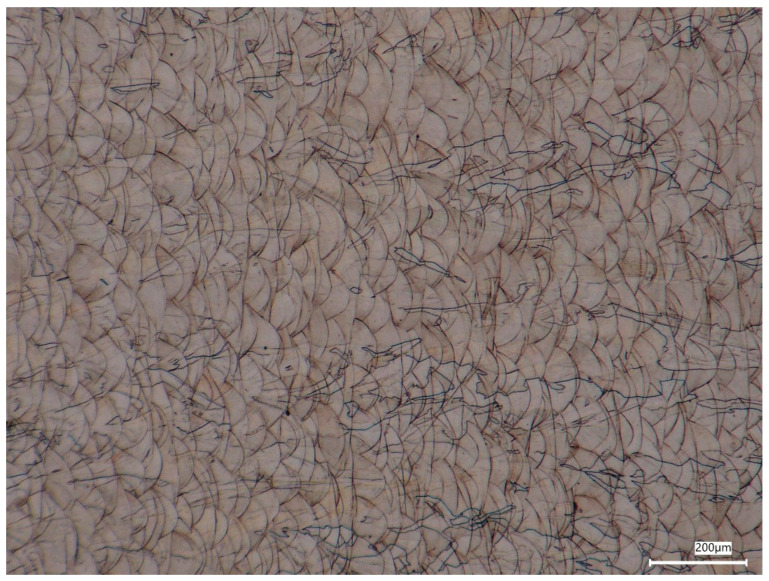
Optical micrograph of DMLS-processed 316L stainless steel showing the characteristic overlapping melt pool structure with a fish-scale/fan-shaped morphology and columnar austenitic grains oriented predominantly perpendicular to the layer interfaces.

Optical microscopy observations of the 316L stainless steel produced by the DMLS process revealed a well-developed layered microstructure typical of laser powder bed fusion technologies. The observed morphology results from the sequential deposition and remelting of successive powder layers during the manufacturing process. The microstructure is dominated by characteristic arc-shaped melt pool boundaries forming a repetitive overlapping pattern often described as a fish-scale or fan-shaped morphology (Figure 6). The melt pools exhibit a semi-elliptical geometry elongated along the laser scanning direction, with clear overlap between adjacent scan tracks. This morphology is associated with the hatch overlap and repeated thermal cycling of previously solidified material during subsequent laser passes, which is commonly reported for additively manufactured 316L stainless steel [28]. Based on the measurements performed using the scale bars in the optical micrographs, the effective height of individual melt pool layers was estimated to be approximately 30–45 µm, while the melt pool width ranged from about 80 to 120 µm. These values are consistent with the literature data for 316L processed with DMLS, where the nominal layer thickness is typically around 40 µm and the apparent layer morphology may vary depending on processing parameters [25,29].

**Figure 7:**
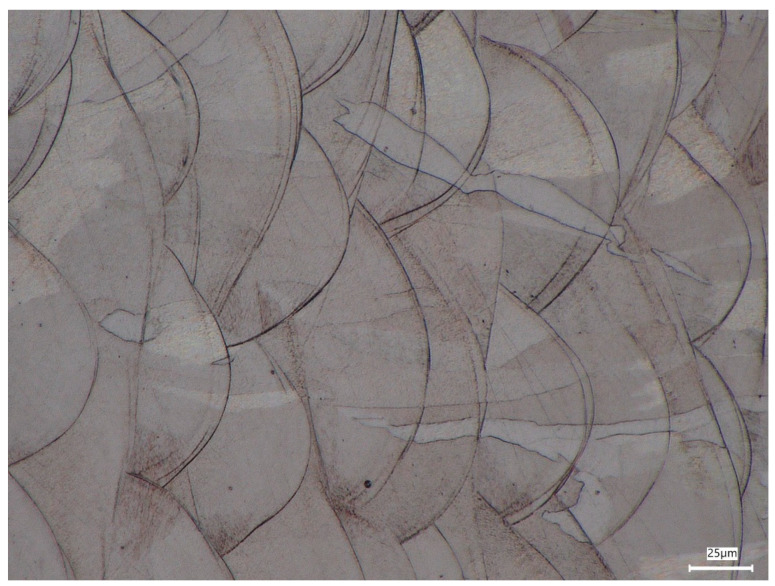
Higher magnification view of the region marked in Figure 6, revealing the semi-elliptical melt pool boundaries and elongated columnar austenite grains extending across adjacent deposited layers.

Within the melt pool structure, elongated austenitic grains are visible (Figure 6 and Figure 7). The grains exhibit an anisotropic columnar morphology and frequently extend across multiple deposited layers. Their preferred growth direction is approximately perpendicular to the layer interfaces, which corresponds to the primary heat flow direction during solidification. This behaviour indicates epitaxial grain growth typical of rapidly solidified DMLS materials [25]. The characteristic austenite grain size estimated from the optical images is in the range of approximately 15–40 µm, locally reaching values close to 50 µm. These values fall within the typical range reported for DMLS-processed 316L stainless steel (~10–50 µm) [26] and are also consistent with EBSD-based equivalent grain diameters reported in the literature [28].

Overall, the examined microstructure appears to be continuous and well consolidated, with a regular melt pool pattern and without visible lack-of-fusion defects at the optical microscopy scale, indicating stable processing conditions during the DMLS manufacturing process.

**Figure 8:**
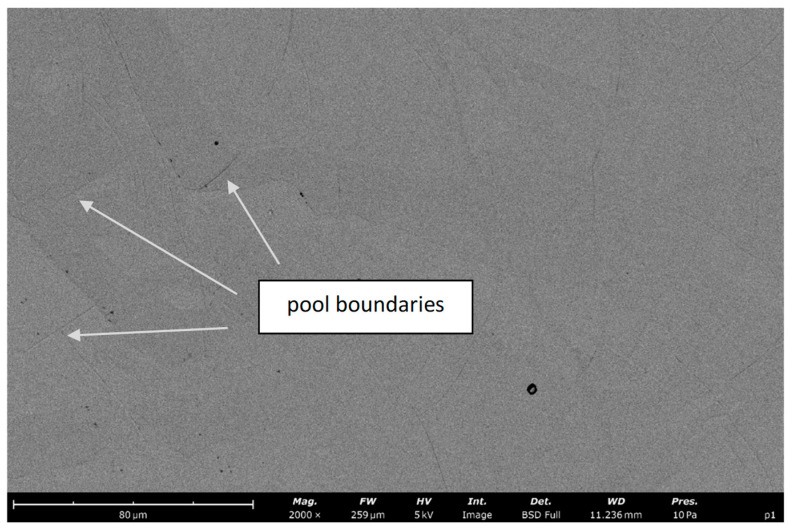
SEM micrograph of DMLS-processed 316L stainless steel showing the austenitic matrix with visible melt pool boundaries and uniformly distributed fine cellular substructure within the grains.

**Figure 9:**
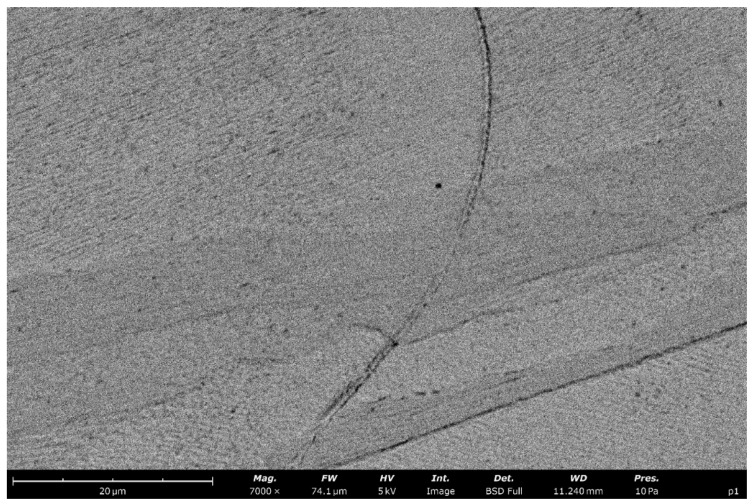
Area enlarged from Figure 8, showing the dense cellular/needle-like substructure within austenite grains, characteristic of rapid solidification during the DMLS process.

SEM observations (BSE detector) of the 316L stainless steel produced by the DMLS process revealed a multiscale hierarchical microstructure characteristic of austenitic alloys manufactured by laser powder bed fusion (Figure 8). At the grain scale, an austenitic matrix and distinct melt pool boundaries are visible, while the grain interiors exhibit a very fine cellular/needle-like substructure (Figure 9). This substructure forms a dense network of dislocation cells with a directional arrangement related to the solidification conditions within the melt pool. Its formation is a consequence of extremely high cooling rates on the order of ~10^6^ K/s and the steep thermal gradients inherent to the DMLS process [27,28]. Based on SEM image analysis, the characteristic size of the cellular features was estimated to be approximately 0.5–1.0 µm, which is in very good agreement with the data in the literature for additively manufactured 316L stainless steel [27,29]. In many regions, bundles of parallel cells/needles with a high aspect ratio are observed, consistent with the morphology widely reported for SLM materials. According to the literature, such a refined cellular substructure combined with a high dislocation density constitutes one of the primary strengthening mechanisms in DMLS 316L stainless steel [27,28,29]. The observed microstructure is uniform and does not reveal extensive lack-of-fusion defects at the SEM scale, indicating stable processing conditions and high consolidation quality of the material.

**Figure 10:**
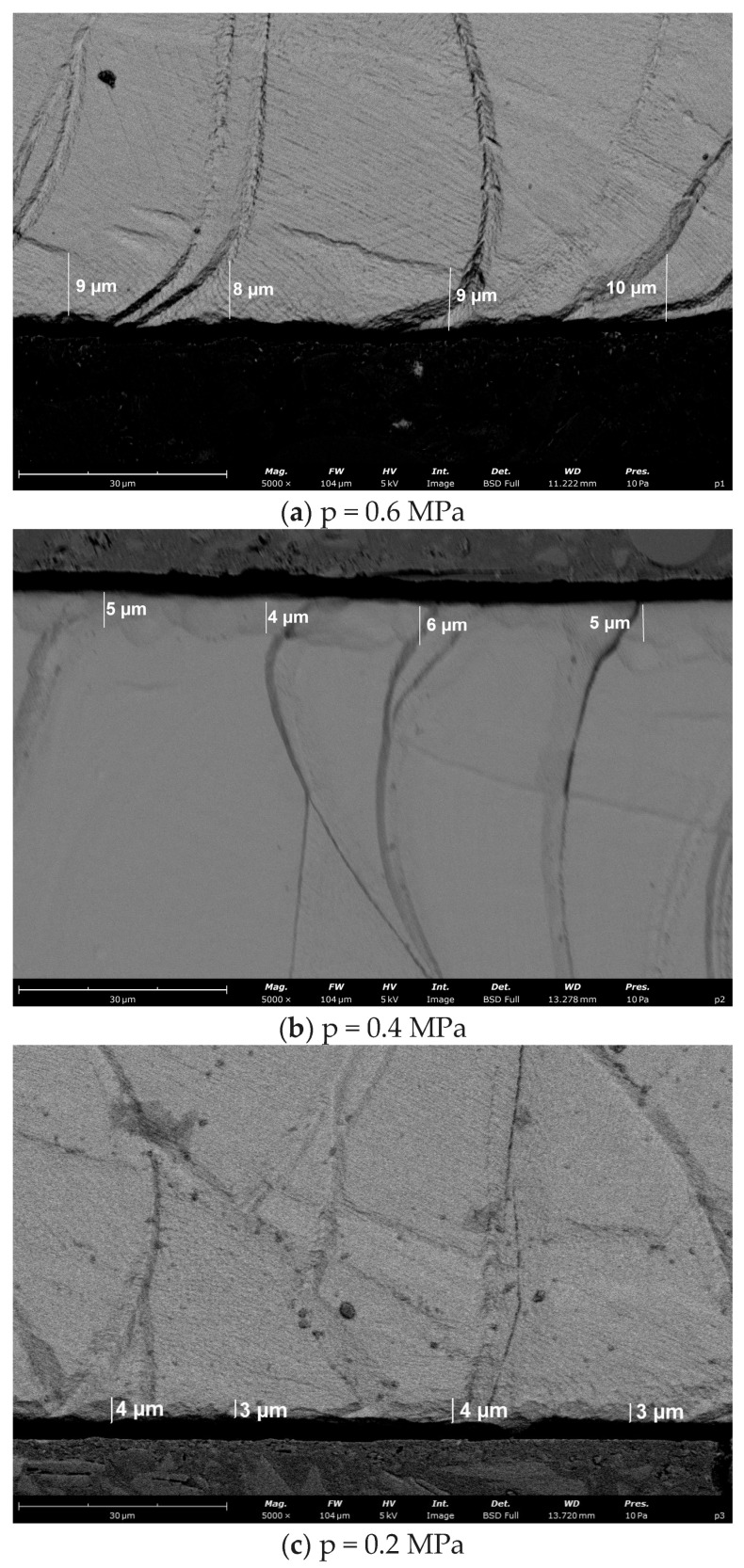
SEM micrographs showing the effect of air supply pressure on the thickness of the strain-hardened surface layer in DMLS-processed 316L stainless steel: (**a**) 0.6 MPa, (**b**) 0.4 MPa, and (**c**) 0.2 MPa (shot diameter 3 mm).

Based on the SEM observations and the performed measurements, a clear influence of shot peening on the near-surface layer of DMLS-processed 316L stainless steel can be confirmed. In the SEM cross-sections of the shot-peened specimens, pronounced effects of shot impact are visible in the near-surface region. The surface edge is not perfectly linear; instead, local waviness and small microirregularities are observed, which are traces of plastic deformation induced by repeated shot impacts. At a constant shot diameter of 3 mm, the depth of plastic deformation is strongly governed by the air supply pressure (Figure 10).

For the highest investigated pressure of 0.6 MPa, a continuous plastically deformed layer with an average thickness of approximately 8 µm (locally up to ~10 µm) is present directly beneath the surface (Figure 10a). Within this zone, the characteristic cellular substructure of the DMLS material appears blurred and locally densified, indicating an increased dislocation density caused by shot peening. In some regions, local bending of the cellular features toward the surface is observed, which is a typical manifestation of strain hardening. As the air supply pressure decreases, the deformation effect becomes progressively weaker. At 0.4 MPa, the thickness of the plastically affected layer is approximately 5 µm (locally ~4–6 µm), and the modification of the cellular substructure is less pronounced (Figure 10b). For the lowest pressure of 0.2 MPa, the deformed zone is thin and weakly developed, reaching only about 3.5 µm on average (on the order of 3–4 µm), and the microstructure rapidly returns to the regular morphology typical of the DMLS material within a few micrometres from the surface (Figure 10c). This monotonic trend confirms that the air supply pressure is a key parameter that controls the depth of plastic deformation and the degree of work hardening.

**Figure 11:**
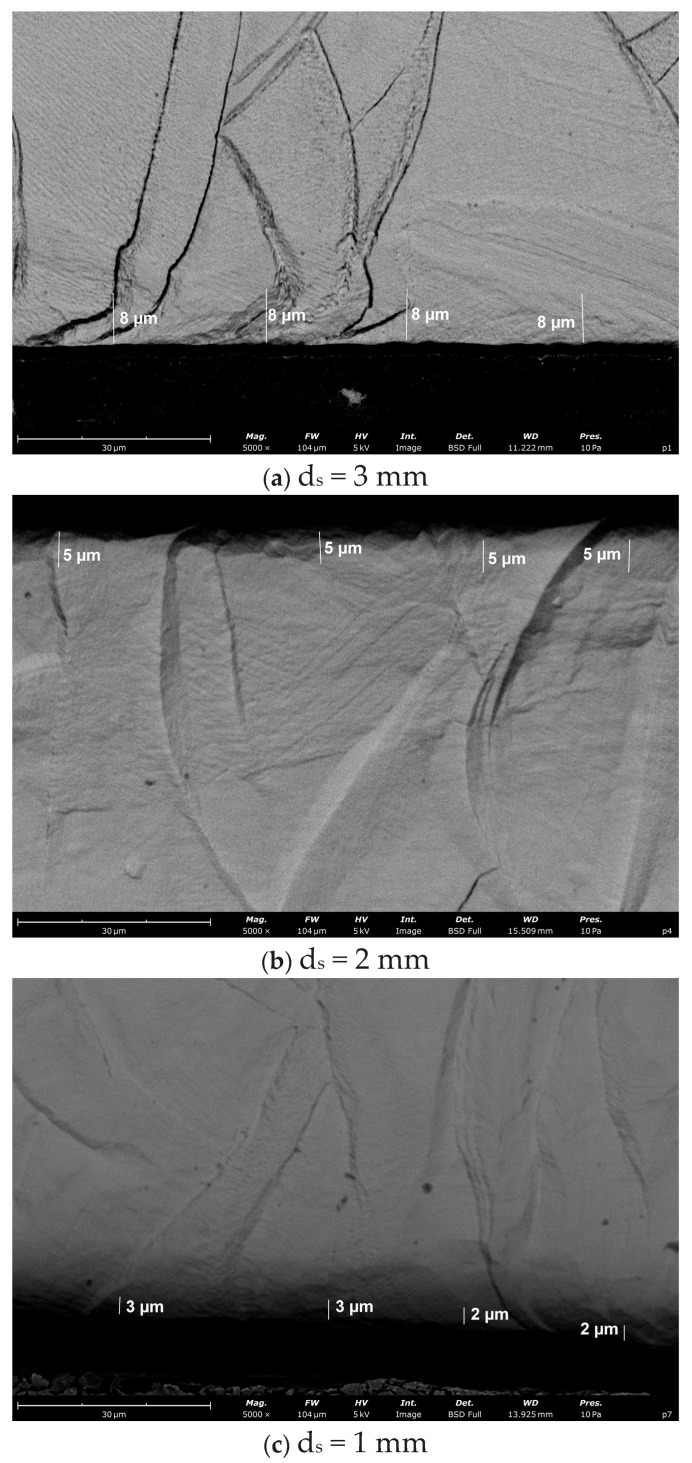
SEM micrographs showing the effect of the shot diameter on the thickness of the strain-hardened surface layer in DMLS-processed 316L stainless steel: (**a**) 3 mm, (**b**) 2 mm, and (**c**) 1 mm (air supply pressure 0.6 MPa).

In addition, a significant effect of shot diameter was observed at a constant pressure of 0.6 MPa. As the diameter of the shot decreased from 3 mm to 2 mm and further to 1 mm, a systematic reduction was observed in the depth of the plastically affected zone and the degree of distortion of the cellular substructure (Figure 11). The strongest modification was obtained for the 3 mm shot (~8 µm), while for 2 mm, the deformed layer was approximately ~5 µm and for 1 mm only ~2–3 µm. This indicates that a larger shot diameter, at the same air supply pressure, produces a stronger impact effect and deeper surface work hardening. Below the near-surface zone, regardless of the peening parameters, the microstructure retains the typical features of DMLS-manufactured material, including clearly visible melt pool boundaries and a regular cellular substructure within austenite grains. No microcracks or signs of delamination were detected on the surface in any of the analysed variants, indicating that the applied shot peening parameters produced controlled and beneficial surface deformation without interruption of material continuity. The observed plastically deformed surface layer after shot peening is consistent with the literature reports for laser powder bed-fused 316L stainless steel. Numerous studies indicate that shot peening of additively manufactured 316L introduces a near-surface work-hardened zone characterised by increased dislocation density, local distortion of the cellular substructure, and the development of compressive residual stresses [30]. Rautio et al. demonstrated that severe shot peening of LPBF 316L produced significant plastic deformation within the subsurface region together with pronounced compressive residual stresses extending to depths of the order of tens to hundreds of micrometres [30]. Similar observations of surface work hardening and microstructure modification after mechanical surface treatments of AM 316L have also been reported in other studies [31]. Therefore, the plastically affected layer identified in the present study—without evidence of surface cracking or delamination—confirms that the applied shot peening parameters induce beneficial surface strengthening while preserving material integrity.

### 3.2. Residual Stresses Analysis

**Figure 12:**
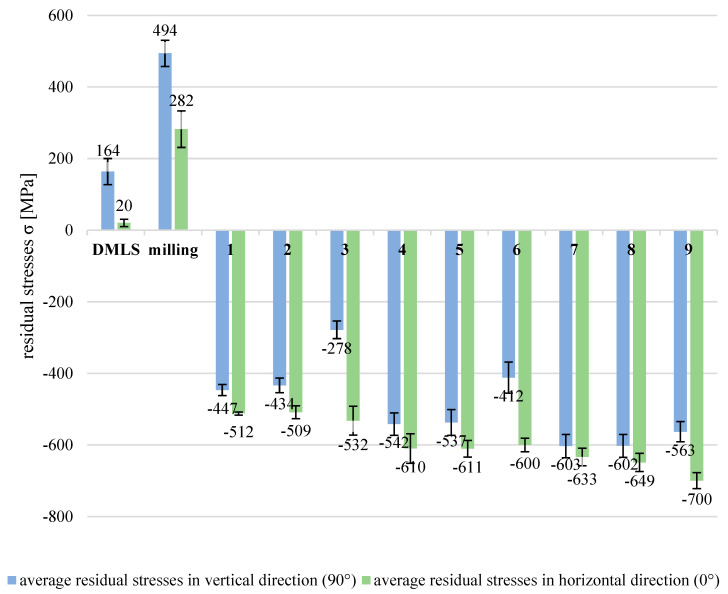
Average values of residual stresses in directions: horizontal (0°) and vertical (90°) after different technological processes and process parameters of shot peening.

Figure 12 presents the average values of the residual stresses in the horizontal (0°) and vertical (90°) directions of direct metal laser-sintered, milled, and shot-peened samples.

The residual stresses of AM components with the application of the DMLS technique characterised a significant anisotropy. In both directions, tensile residual stresses were found, but they were several times greater in the vertical direction corresponding to the built direction of the sample than they were in the horizontal orientation. The unfavourable residual stress state is a quite typical problem for additively manufactured metal elements and is reported by other researchers [3,4,16,21,22,29,30]. The residual stress state worsened substantially after the milling process. The average value of tensile residual stresses reached almost 500 MPa in the vertical direction, while in the horizontal section, it was approximately 282 MPa. Such large tensile residual stresses negatively influence the mechanical properties and may cause component damage during operation. The application of the shot peening process after the milling process changed the residual stress state completely. Regardless of the technological parameters of shot peening, the values of residual stresses became compressive. Generally, the smallest shot diameter (d_s_ = 1 mm) is the greatest value of compressive stresses. The largest average value of the compressive residual stresses (−700 MPa) was obtained at the smallest air supply pressure of 0.2 MPa and was found in the horizontal direction. The satisfactory compressive residual stresses were noticed in the vertical direction after shot peening, but the values were smaller than those in the horizontal direction. The huge difference in average compressive stresses was achieved in series processed with the biggest shot diameter (d_s_ = 3 mm) and the smallest air supply pressure (p = 0.2 MPa). In the horizonal section, the residual stresses (−532 MPa) were approximately twice greater in comparison to those in the vertical section (−278 MPa). Because of this difference in the effect of the shot peening process on the residual stress values, an additional analysis was introduced to find out how shot peening parameters influenced the residual stresses separately in the horizontal and vertical directions. In Table 3, the results of residual stress measurements in both directions after the shot peening process in nine variants are presented.

The results obtained were subjected to the statistical analysis to check if the repeatability of the test conditions was fulfilled. The Cochran test was applied to assess these criteria, and the satisfactory repeatable conditions of the test for both directions of the residual stresses were confirmed. To check the significance of the response effects (coefficients), the *t*-Student test was used. The significance level in the study was set to 5% (α = 0.05). The effect size of the coefficients represented by the strength of the effect on the residual stresses in the horizontal direction is shown in Table 4.

**Figure 13:**
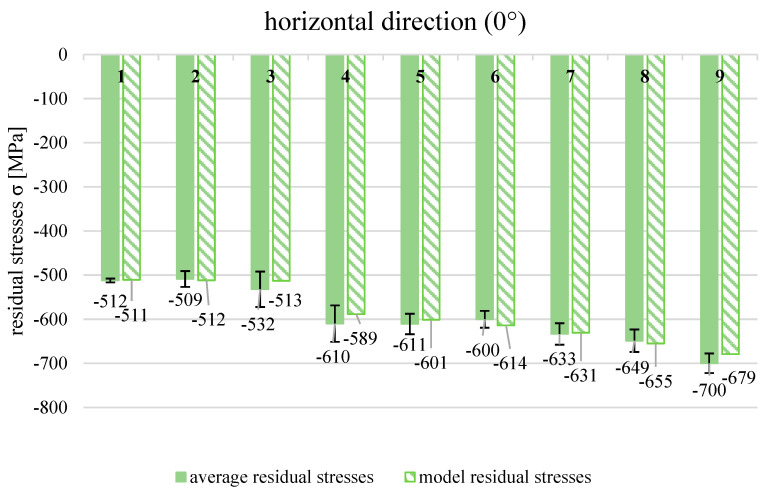
Average and model values of residual stresses in horizontal direction (0°) after shot peening with different parameter values.

The response model with significant coefficients enabled us to calculate the model values that are predicted by the obtained formula. To assess the conformity of the model to experimental results, the Fisher–Snedecor test was used. The model was found to fit the experiment very well (F = 1.69 < F_t_ = 2.93). The graphic representation of the experimental and model values of residual stresses after shot peening (horizontal direction) is shown in Figure 13.

The model values calculated from the response function are within the range of the standard deviation of the average values of the residual stresses obtained from the experiment. The effects of the shot diameter on the residual stresses are linear, nonlinear (quadratic), and also interactive. Evidently, the biggest effect the shot diameter has is in a linear manner. The quadratic effects of the shot diameter and the term of interaction with the air supply pressure are very little on the residual stresses in the horizontal direction but, according to statistical analysis, are significant. One effect is statistically not significant, but it corresponds to the quadratic effect of the air supply pressure on the residual stresses (in the horizontal direction). Generally, the effects of air supply pressure are only slightly or not significant on residual stresses in the horizontal direction of the 316L stainless steel after shot peening in the assumed technological parameter range. In principle, it could be summarised that the main technological parameter that influences strongly the residual stresses in the horizontal direction is the diameter of the shot. The effect of air supply pressure is statistically important but only a little. With the application of the smallest shots, the greatest compressive residual stresses were induced, which is in good agreement with the literature [21,30]. The shot peening with usage of the shots of 0.40–0.85 mm in size caused approximately −800 MPa of compressive residual stresses in the surface layer of SLS 316L stainless steel [21]. These slightly greater compressive residual stresses achieved by Gundgire et al. might be the result of setting a higher pressure of 0.74 MPa during the process.

Because of characteristic anisotropy in the microstructure of DMLS components, it is worth comparing the effects in the vertical direction (corresponding to the built direction) on the state of residual stresses. The effect size of the coefficients represented by the strength of the effect on the residual stresses in the vertical direction is presented in Table 5.

**Figure 14:**
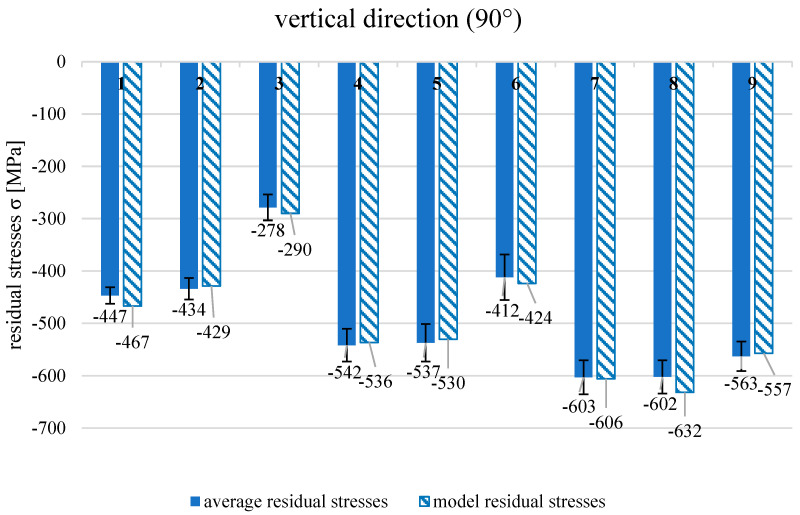
Average and model values of residual stresses in vertical direction (90°) after shot peening with different parameters values.

The response equation with significant effects was used to calculate the model values of the residual stresses in the vertical direction. The conformity of the response equation was checked, and the model obtained fit the experiment very well (F = 1.38 < F_t_ = 2.93). The graph containing the experimental and model values of residual stresses after shot peening (vertical direction) is shown in Figure 14.

The effect of air supply pressure has a completely different impact on residual stresses in the vertical direction than in the horizontal one. In the case of residual stresses in the vertical direction, the effects of air supply pressure are linear, quadratic, and interactive. The strength of the effects is also several times greater than that of the residual stresses in the horizontal direction. In addition, the sign of coefficients corresponding to the air supply pressure of linear effects is negative and that of quadratic effects is positive. As a result of such a relationship, with the increase in air supply pressure, the compressive residual stresses in the vertical direction are greater, but this increase decreases with the decrease in shot diameter. The strong effect of the air supply pressure corresponds with the visible plastic deformation differences in the cross-sections. After shot peening at 0.6 MPa (Figure 10a), the increase in dislocation density is observed, whereas quite small changes in the microstructure are seen after shot peening at 0.2 MPa (Figure 10c). The role of the diameter of the shots is also important because the application of the smallest shots (d_s_ = 1 mm) in the shot peening process resulted in the greatest compressive residual stresses, and their values then were not so sensitive to the air supply pressure. To assure the appropriate residual stress state of the additively manufactured components fabricated with the DMLS technique, one should focus on the residual stress value in the built direction. In this direction, DMLS components are characterised by greater tensile residual stresses than in the horizontal direction, and they influence the residual stresses state after successive technological processes. The relationships between the residual stresses and the technological parameters are more complex in the vertical (built) direction in comparison to those in the horizontal one. The selection of technological parameters of shot peening for the DMLS components should include the assessment of effects especially in the built direction: firstly, because in the vertical (built) direction the compressive residual stresses are smaller than in the horizontal direction, and secondly, because the results are much more sensitive to changes in the technological parameters than in the horizontal direction. The residual stress values obtained agree well with other authors’ results [21,22,29,30], who also achieved the favourable compressive residual stresses after shot peening the surface of AM 316L stainless steel.

**Figure 15:**
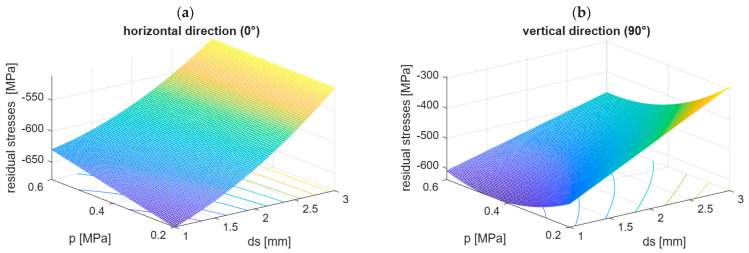
Plots of the model values of residual stresses in the directions: horizontal (0°) (**a**) and vertical (90°) (**b**) versus the process parameters of shot peening in the examined ranges.

The strength effects were analysed on coded parameters, and for some practical aspects, the plots of predicted residual stresses versus the technological parameter settings are presented in Figure 15.

The shape of the plot of the residual stresses in the horizontal direction (0°) is confirmation that the diameter of the shots d_s_ influenced the results the most and that the air supply pressure p influenced the residual stresses but not so intensely. Based on this plot, the maximum compressive residual stresses might be obtained with the smallest shot (d_s_ = 1 mm) application and also the smallest air supply pressure (p = 0.2 MPa) during the shot peening process. The shape of the plot of the residual stresses in the vertical direction (90°) is more variable, which confirms more complex relationships between residual stresses and technological parameters. The shot diameter influenced the results, but the air supply pressure strongly influenced the residual stresses in linear, quadratic, and interactive manners. The greatest compressive residual stresses were found with the smallest shot (d_s_ = 1 mm) usage and a medium air supply pressure (p = 0.4 MPa). Differences in residual stress values in the horizontal and vertical directions confirmed the anisotropy of this feature in the surface layer of AM 316L stainless steel after shot peening. But maybe more attention should be paid to DMLS technology parameter selection and their optimisation because the difference in the residual stresses of as-built samples differed significantly in the horizontal and vertical directions. The microstructure is characterised by anisotropy but is assessed as fine and almost without defects. It seems to be a space not only to develop the post-processing technology like shot peening but also to improve the additive manufacturing techniques in the aspect of more isotropic properties (of residual stresses) or at least in the directions that the loads are attached.

### 3.3. Surface Topography Analysis

**Figure 16:**
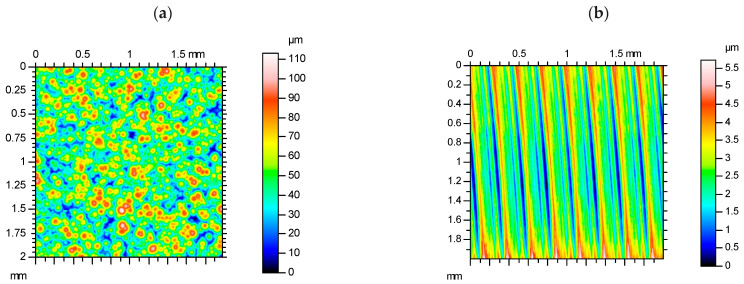
Axonometric views of the direct metal laser-sintered (**a**) and milled (**b**) surfaces of AM 316L stainless steel.

In Figure 16, the axonometric views of DMLS of 316L stainless steel as is built and the same type of component but after the milling process are presented.

The morphologies of the surfaces are entirely dissimilar. While the additively manufactured element is characterised by zones with melted material in the form of rounded islands with random distribution, the milled surface has regular almost straight paths reflecting the passage of the tool. The DMLS component looks quite typical, as it has a surface morphology of characteristic isotropy of the element arrangement. At the same time, the typical pattern of the laser path is not visible in Figure 16a.

The surface topography parameters gave more detailed information about the compared surfaces (Table 6). The surface of DMLS 316L as built is characterised by high average surface parameter Sa but is in the limits of this process. The maximum height of the peaks Sp is of comparable size as the diameter of metal particles that were in the sintering process. That may suggest that there are unmelted or not fully melted particles. The surface parameter corresponding to the deepest valley Sv is quite large, but the height of individual peaks is greater, which is also confirmed by positive skewness Ssk. The asymmetry of peaks and valleys also means that not only do individual peaks stand out from other irregularities but there are more peaks than valleys. Kurtosis of the surface of 316L as built is close to a Gaussian distribution, with a slight inclination to a platykurtic distribution pattern. The parameter of surface texture Str is close to 1, which is characteristic of an isotropic surface, so the anisotropy in surface texture after the DMLS process was not found using this technique. Generally, the height surface parameters were large although in the expected assumptions. The decrease in the surface parameter values by shot peening is limited. Some results found in the literature report a great reduction in surface roughness of up to about 60% [21,24], but sometimes surface irregularities increased after shot peening the AM surface [19]. In the present study, the surface topography is of large height, and the milling process was used to obtain the surface of the Sa parameter below 1 µm.

**Figure 17:**
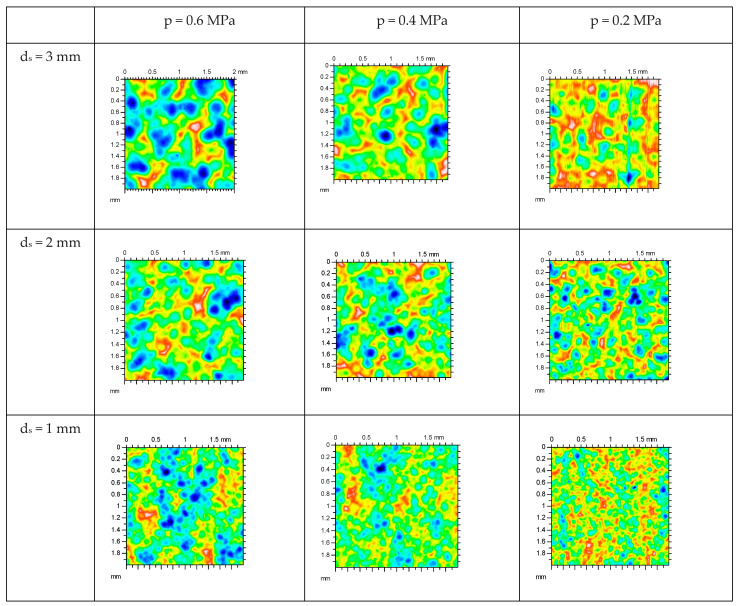
Axonometric views of the exampled surfaces of sintered 316L stainless steel after milling and shot peening with different technological parameters.

The reduction of almost all amplitude parameters after milling process is significant. The arithmetical mean height of the milled surface Sa is at a quite fine level. Skewness Ssk of the milled surface means almost perfect symmetry, and kurtosis is very close to normal distribution. The evident anisotropy of the milled surface was confirmed by parameter Str, which is close to 0. Because the milling process introduced large tensile residual stresses, the surfaces of 316L stainless steel were subjected to shot peening, and their surface topography was also analysed. Figure 17 presents the axonometric views of the example sintered surfaces after milling and shot peening with different technological parameters. The surface topography of all the presented samples is like that of typical shot-peened surfaces. There are randomly placed nearly circular spots caused by the shots’ impact, and around them, there is material flash. The greatest spots are observed on surfaces after shot peening with the greatest shot diameter and the biggest air supply pressure, but after decreasing the air supply pressure to 0.2 MPa and with shots of 3 mm, the plastic deformation was not so intense. In this case, the straight paths of the milling tool from pre-treatment are still visible. This variant was characterised by the smallest compressive residual stresses (−278 MPa in the vertical direction) from all the shot-peened series. There are no more surface topographies where the milling marks remained in such an evident way. With the decrease in shot diameter, the size of spots decreases too, but the intensity of shot peening seems to be greater. For the smallest air supply pressure, the coverage degree of the shot peening marks is the greatest.

Surface topography parameters of all shot-peened series are shown in Table 7. The quantitative measures brought more light onto the surface topography results obtained after the shot peening process.

The shot peening process caused an increase in all analysed height parameters. The highest surface irregularities were found at the highest air supply pressure of 0.6 MPa. The average (Sa) and maximum (Sp and Sv) surface amplitude parameters were more than twice that after milling. The skewness of the shot-peened surfaces processed at an air supply pressure of 0.6 MPa is also the greatest, which means that the asymmetry with dominant peaks occurs. Negative skewness was obtained for series with shot diameters of 3 mm and 2 mm when the air supply pressure was the smallest (0.2 MPa), which corresponded to the dominant valleys left after milling. The milling paths in surfaces of series no. 3 were very visible in the axonometric views. The remaining paths after milling tool cutting on the surface of series no. 6 was not so obvious, especially that the surface texture parameter was the largest (Str = 0.896), which is characteristic of an isotropic surface. This series (no. 6) was also characterised by the smallest compressive residual stresses in the built direction in group of series processed with shots of 2 mm in diameter. Generally, the smallest values of surface height parameters were achieved with the application of the smallest shots (d_s_ = 1 mm) and the smallest air supply pressure (p = 0.2 MPa), and these values were similar to those of milling surfaces. Skewness Ssk and kurtosis Sku values were similar to the Ssk and Sku parameters of the milling surface. Interesting results of surface texture Str were found for the shot-peened surfaces processed with the smallest shots because the value of Str is not as for isotropic surfaces, although the milling paths are not visible and negative skewness is not noted. It is probable that the small shots hit the complete surface and flatten the irregularities, but waviness as a remaining of the milling process still existed, and the mixed character of the surface texture is observed. The smallest shots are also of the lowest individual masses, and the energy that they hit the surface with is significantly lower in comparison to other shots that were bigger and heavier. It seems that a very important factor is the plastic deformation of the individual surface irregularities with the small shots that reach every location. The shot peening process with application of small shots resulted in the greatest compressive residual stresses, even at the smallest air supply pressure of 0.2 MPa. The thickness of plastic deformation in the surface layer after shot peening with small shots (d_s_ = 1 mm) was slight, approximately of a few microns, but taking into account the combined effect of the minimal surface topography height and maximum compressive residual stresses after shot peening, these shots should be applied to process the surface of AM 316L stainless steel. Small shot applications were also very effective in introducing high compressive residual stresses in the vertical direction, which corresponds with the built direction of the DMLS components. In experiment nos. 6-9, even at the lowest air supply pressure of 0.2 MPa, the compressive residual stresses were quite high. Comparing results in all conducted experiments, only with an air supply pressure of 0.2 MPa was the surface height low and comparable to those after milling. With a higher air supply pressure of 0.4–0.6 MPa, the plastic deformation of the surface was extensive and caused an increase in the height parameters of the surface topography.

For statistical analysis, the parameter of the mean arithmetical surface height Sa was selected. The repeatability of the test conditions was satisfied. The effect size of the coefficients representing the strength of the effect on the mean arithmetical surface height Sa is shown in Table 8.

**Figure 18:**
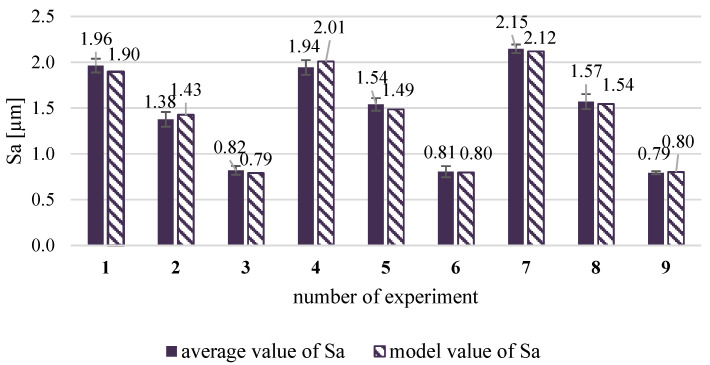
Average and model values of mean arithmetical height of the surface Sa after shot peening with different parameters values.

The model values were calculated using the response model with the significant coefficients. The conformity of the obtained model to the experimental results was assessed with the Fisher–Snedecor test. The model fit the experiment very well (F = 2.917 < F_t_ = 2.93). The graphic representation of the experimental and model values of the mean arithmetical surface height Sa is shown in Figure 18.

The effect of the air supply pressure was of the greatest strength on the mean arithmetical height of surface Sa in the shot peening process. The effects of air supply pressure are linear, quadratic, and interactive, but the linear relationship was definitely several times greater than the nonlinear and interaction ones. The shot diameter had the most significant effect on the residual stresses, but in the case of its effect on the mean arithmetical height of surface Sa, the shot diameter had quite small linear and interaction effects and not significant quadratic ones. Contrary to the relationships with residual stresses, where the shot diameter was the most important parameter, the surface height is the most sensitive to the air supply pressure. The air supply pressure equal to 0.2 MPa during the shot peening process resulted in small surface irregularities, and the mean surface height was similar to Sa value obtained after milling process. According to the literature, the surface roughness of as-built elements with the additive manufacturing technique acted as the stress concentrator for the fracture initiation [15] and needs optimisation according to the operating conditions of the AM elements. Surface roughness and residual stresses are crucial factors in mechanical components that influence fatigue strength or corrosion resistance [10,11]. There are results that the surface roughness of AM elements was reduced due to shot peening [9,21,22], but the milling process is an option to substantially reduce the surface height. Eventual tensile residual stresses induced during the milling process might be reduced and converse into compressive with shot peening, as is demonstrated in this study.

## 4. Conclusions

In this study, the surface layer of DMLS 316L stainless steel was examined as built, after milling, and after shot peening. The surface of AM 316L stainless steel was characterised by typical large irregularities in the form of rounded islands after powder melting and tensile residual stresses that are detrimental for the metal elements’ performance. As the results of conducted experiments and analysis, some findings were indicated:

- The application of both consecutive milling and shot peening processes successfully improved the surface layer quality of DMLS 316L stainless steel by reducing the surface topography height and the introduction of compressive residual stresses.
- The low surface topography height obtained after milling was maintained at a similar level after shot peening with the use of the smallest shots of 1 mm and the smallest air supply pressure equal to 0.2 MPa.
- Shot peening diminished not only the tensile residual stresses after the DMLS process but also large tensile residual stresses after milling and changed them into compressive ones. For maximising the compressive residual stresses, due to shot peening the DMLS 316L stainless steel, the smallest shots of 1 mm are recommended, but the setting of the air supply pressure depends on the load direction.
- The residual stresses in all examined samples—direct metal laser sintered, milled, and shot peened—were characterised by high anisotropy. Smaller tensile residual stresses of direct metal laser-sintered and milled materials were in the orientation perpendicular to the built direction than the residual stresses along the built direction. Shot peening induced compressive residual stresses, but the difference value in directions remained similar to the hereditary nature of the successive technologies.

## Figures and Tables

**Table 1:** Chemical composition of 316L stainless steel after the DMLS process.

Chemical Element	Fe	Cr	Ni	Mo	C	Mn	Cu	P	S	Si
Wt%	balance	18.6	13.4	2.9	0.01	1.7	0.02	0.01	0.01	0.3

**Table 2:** Technological parameters of DMLS, milling, and shot peening (9 experiments according to the full factorial design 3^2^).

DMLS	Laser Power 200 W, Laser Speed 1080 mm/s, Hatch Distance 0.09 mm, Laser Spot Size 100 µm, and Layer Thickness 20 µm	
Milling	rotational speed 400 rot/min, feed 0.1 mm/rot, and depth 0.1 mm	
Shot peening	Shot diameter d_s_ [mm]	Air supply pressure p [MPa]
Exp. 1	3	0.6
Exp. 2	3	0.4
Exp. 3	3	0.2
Exp. 4	2	0.6
Exp. 5	2	0.4
Exp. 6	2	0.2
Exp. 7	1	0.6
Exp. 8	1	0.4
Exp. 9	1	0.2

**Table 3:** Residual stress values in horizontal (0°) and vertical (90°) directions for DMLS samples after shot peening (milling before shot peening).

Nr of Exper.	d_s_ [mm]	p [MPa]	Residual Stresses in the Horizontal Direction (0°)				Residual Stresses in the Vertical Direction (90°)			
			σ_1_ [MPa]	σ_2_ [MPa]	σ_3_ [MPa]	σ_av._ [MPa]	σ_1_ [MPa]	σ_2_ [MPa]	σ_3_ [MPa]	σ_av._ [MPa]
1	3	0.6	−507	−515	−514	−512	−463	−445	−432	−447
2	3	0.4	−521	−488	−517	−509	−451	−411	−439	−434
3	3	0.2	−550	−486	−560	−532	−290	−250	−295	−278
4	2	0.6	−656	−598	−576	−610	−550	−568	−507	−542
5	2	0.4	−584	−627	−621	−611	−537	−573	−501	−537
6	2	0.2	−622	−588	−590	−600	−443	−430	−362	−412
7	1	0.6	−624	−661	−615	−633	−639	−576	−594	−603
8	1	0.4	−657	−669	−620	−649	−592	−638	−577	−602
9	1	0.2	−713	−712	−674	−700	−589	−567	−533	−563

σ_i_—measured value of residual stresses in i-replication; σ_av._—average value of residual stresses.

**Table 4:** The effect size of the coefficients represented by the strength of the effect on the residual stresses in the horizontal direction (0°).

Response Effects (Coefficients)						
b_0_	b_1_	b_2_	b_11_	b_22_	b_12_	b_t_
−600.22	71.50	12.72	17.83	−8.50	−11.58	10.70
significant	significant	significant	significant	not significant	significant	test value

**Table 5:** The effect size of the coefficients represented by the strength of the effect on the residual stresses in vertical direction (90°).

Response Effects (Coefficients)						
b_0_	b_1_	b_2_	b_11_	b_22_	b_12_	b_t_
−530.30	101.61	−56.39	8.94	50.28	−32.08	12.30
significant	significant	significant	not significant	significant	significant	test value

**Table 6:** Surface topography parameters of direct metal laser-sintered and milled surfaces.

Parameters	DMLS	Milling	Difference %
Sa [µm]	13.2	0.741	−94.4
Sp [µm]	64.1	3.2	−95.0
Sv [µm]	49.0	2.53	−94.8
Ssk [-]	0.257	0.0127	−95.1
Sku [-]	2.87	2.78	−3.1
Str [-]	0.944	0.033	−96.5

**Table 7:** Surface topography parameters of shot-peened surfaces and after milling.

Nr of Experiment	d_s_ [mm]	p [MPa]	Sa [µm]	Sp [µm]	Sv [µm]	Ssk [-]	Sku [-]	Str [-]
0	milling		0.741	3.2	2.53	0.013	2.78	0.033
1	3	0.6	1.963	8.47	5.41	0.397	2.76	0.791
2	3	0.4	1.377	6.08	5.24	0.017	3.23	0.771
3	3	0.2	0.820	3.27	4.28	−0.166	2.89	0.590
4	2	0.6	1.943	8.99	7.18	0.132	3.04	0.880
5	2	0.4	1.540	6.88	5.97	0.145	2.94	0.795
6	2	0.2	0.805	3.79	3.30	−0.012	2.89	0.896
7	1	0.6	2.147	10.60	7.64	0.235	2.89	0.369
8	1	0.4	1.570	9.40	7.65	0.183	3.34	0.402
9	1	0.2	0.793	3.88	3.95	0.091	2.82	0.292

**Table 8:** The effect size of the coefficients represented the strength of the effect on the mean arithmetical surface height Sa.

Response Effects (Coefficients)						
b_0_	b_1_	b_2_	b_11_	b_22_	b_12_	b_t_
1.485	−0.058	0.606	0.015	−0.083	−0.052	0.026
significant	significant	significant	not significant	significant	significant	test value

## Data Availability

The original contributions presented in this study are included in the article material. Further inquiries can be directed to the corresponding author.
